# Conflicting findings between the Mitra-Fr and the Coapt trials: Implications regarding the cost-effectiveness of percutaneous repair for heart failure patients with severe secondary mitral regurgitation

**DOI:** 10.1371/journal.pone.0241361

**Published:** 2020-11-09

**Authors:** Xavier Armoiry, Jean-François Obadia, Peter Auguste, Martin Connock

**Affiliations:** 1 Warwick Medical School, University of Warwick, Coventry, United Kingdom; 2 School of Pharmacy (ISPB)/UMR CNRS 5510 MATEIS/Edouard Herriot Hospital, Pharmacy Department, University of Lyon, Lyon, France; 3 Hôpital Cardiovasculaire Louis Pradel, Chirurgie Cardio-Vasculaire et Transplantation Cardiaque, Hospices Civils de Lyon and Claude Bernard University, Lyon, France; Icahn School of Medicine at Mount Sinai, UNITED STATES

## Abstract

**Purpose:**

Two randomized controlled trials (RCTs), Mitra-Fr and Coapt, evaluating the benefit of percutaneous repair (PR) for heart failure (HF) patients with severe mitral regurgitation, have led to conflicting results. We aimed to evaluate the impact of these trial results on the cost-effectiveness of PR using effectiveness inputs from the two RCTs.

**Methods:**

We developed a time varying Markov type model with three mutually exclusive health states: alive without HF hospitalisation, alive with HF hospitalisation, and dead. Clinically plausible extrapolations beyond observed data were obtained by developing parametric modelling for overall survival and HF hospitalisations using published data from each trial. We adopted the perspective of the French Health System and used a 30-year time horizon. Results were expressed as € / quality-adjusted life year (QALY) gained using utility inputs from literature.

**Findings:**

Results are presented using treatment efficacy measures from Mitra-F and Coapt trials respectively. With the Mitra-Fr data, after annual discounting, the base case model generated an incremental 0.00387 QALY at a cost of €25,010, yielding an incremental cost effectiveness ratio (ICER) of €6,467,032 / QALY. The model was sensitive to changes made to model inputs. There was no potential of PR being cost-effective.

With the Coapt data, the model generated 1.19 QALY gain at a cost of €26,130 yielding an ICER of €21,918 / QALY and at a threshold of >€50,000/QALY PR had a probability of 1 of being cost-effective.

**Implications:**

Cost effectiveness results were conflicting; reconciling differences between trials is a priority and could promote optimal cost effectiveness analyses and resource allocation.

## Introduction

Percutaneous repair (PR) with the Mitraclip system is a breakthrough technology which was developed to improve clinical outcomes in patients with severe mitral regurgitation (MR) [1].

Although the expansion of PR has not followed the exponential growth seen with transcatheter aortic valve implantation (TAVI) [2], it is increasingly used and the cap of 100,000 Mitraclip implantations worldwide was reached late September 2019 [3].

While the first randomised controlled trial (RCT) evaluating this medical device mainly enrolled patients with primary MR [4], there has been a shift over time in the indications to patients with secondary MR [5, 6]. This trend was in contrast to the level of evidence supporting the clinical benefit of percutaneous repair (PR), which was low (class IIb, level C) in the absence of head-to head comparison evaluating efficacy in symptomatic heart failure (HF) patients with severe secondary MR [7].

In 2018, further advances were made with the publication of the first two RCTs comparing clinical outcomes after guideline-directed medical treatment (GDMT) plus PR versus GDMT alone in this severely ill population [8, 9]. The RCT findings were conflicting, with the Mitra-Fr study showing null results on the primary endpoint at 1 year (composite rate of deaths or first rehospitalization for heart failure) [8] while the Coapt study showed positive results on the primary endpoint at 2 years (cumulative rate of rehospitalization) as well as on mortality [9]. At two years follow up of the Mitra-Fr study Iung et al. (2019) [10] confirmed the absence of benefit on the all pre-specified endpoints but also an unpredicted trend in favour of the Mitraclip group beyond one year, which suggests the interest of using modelling technique to predict longer-term outcomes beyond observed data. Such approach could also be relevant to explore if a potential trend towards favourable results on HF hospitalisations could have some impact in terms of reducing costs and/or improving quality of life.

In addition to the uncertainty around the potential benefit of PR, the conflicting findings of the two trials have considerable economic implications given the substantial cost of the device, the use of which is may not be well optimized since no clear explanation has been identified to account for the differences in outcome from the two trials.

Previous cost-effectiveness estimates of the Mitraclip device have analysed data only from the Coapt trial [11] or used propensity matching of retrospective observational data for Italian patients who received medical management with or without addition of Mitraclip surgery [12]. In view of the conflicting and recent results from two randomised trials we aimed to evaluate their impact on cost-effectiveness estimates of PR for heart failure patients with severe secondary MR. We therefore estimated the cost effectiveness of PR + GDMT vs. GDMT alone using data at two years from a] Mitra-Fr and b] Coapt.

## Methods

### Overview

We performed a cost-effectiveness analysis comparing PR with the Mitraclip system (Abbott Vascular) + GDMT versus GDMT alone. We adopted the perspective of the French Health Insurance hypothesizing full reimbursement of expenses associated with PR used in patients with secondary MR. We used a lifetime horizon of 30 years. In the absence of long-term outcomes reported in the literature (maximum follow-up duration with no cross-over being 24 months in both trials), we modelled lifelong costs and clinical outcomes including health-related quality of life.

### Model, population, treatment and effectiveness

We developed a continuous time cohort model (time varying Markov type) with three mutually exclusive health states: alive free of heart failure hospitalisation, alive with heart failure hospitalisation, and dead (S1 Appendix in S1 File).

Markov type modelling is the most common approach used in the literature for HF [13]. Apart of alive/death states and HF hospitalisations states, some published models include further transitions across New York Heart Association (NHYA) disease stages [13] which was not possible in our model due to the lack of available data for the Mitra-fr and Coapt trials. The choice of our health states was also guided by trial information available in the public domain which did not enable to distinguish between death in and out of hospital. However, since all deaths are accounted for in the model at the time at which they were recorded we assume any inaccuracies to be minimal. The model compared the medical cost and health outcomes for two strategies: PR + GDMT versus GDMT alone. The population was a cohort with the same characteristics as those in the Mitra-Fr or Coapt trials [8, 9]. The baseline characteristics on patients enrolled in the two trials have been summarised in S2 Appendix in S1 File. Briefly, Mitra-Fr and Coapt trials have included patients presenting reduced ejection fraction heart failure (HF) with severe secondary mitral regurgitation. The starting age of the population was the same to that in the two trials. The whole cohort was entered into the models in the alive non-hospitalised state. We used a cycle length of one month, consistent with other published models specific to heart failure [14, 15], and a 30-year horizon so as to ensure less than 1% of survivors.

After each cycle, patients could transition to death or to HF hospitalisation. After HF hospitalisation, patients transitioned back to the alive non-hospitalised state.

The monthly probabilities of transition from alive non-hospitalised to death state or to HF hospitalisation were derived from overall survival (OS) and cumulative HF hospitalisation (HFH) curves respectively which were reported in both the Mitra-Fr and Coapt trials [9, 10].

In order to fit parametric models for OS and HFH we digitized the published Kaplan Meier (KM) OS and cumulative HFH plots from the two trials (using Digitizelt software), then for OS we used the method of Guyot et al. [16] to estimate of underlying individual patient data (IPD) and reconstruct KM plots and parametric models of time to event (S2.1 and S3.1 Appendices in S1 File). The number of HFHs at each month were read from digitised HFH plots.

For OS we fitted standard parametric models using Stata version 15.0 (Statacorp, College Station, TX, USA) with the *stgenreg* package [17] and judged goodness of fit by visual inspection and using Akaike’s Information Criteria (AIC) (S2.2 and S3.2 Appendices in S1 File). On the basis of AIC scores and clinical plausibility, the best fit for GDMT OS in the Mitra-Fr study was the exponential model which was used in the base-case. For face validity check, we also examined the predicted OS extrapolations of our model against published curves. The OS model for the Mitraclip arm, was derived from the GDMT arm applying a hazard ratio (HR) of 1 consistent with the absence of survival difference observed in the trial. Indeed, at two years, the rates of death from all cause were 23.1% (PR+GDMT) and 22.8% (GDMT) and the HR was 1.02 (0.70, 1.50) [10]. Moreover, based on visual inspection of the Kaplan Meier curve for OS, there was no clear trend to suggest that curves may separate over time. While we could have used the HR of 1.02 in our analyses as reported in the trial, this would have conferred a slight but artificial benefit on OS for one strategy over the other, which again justifies our choice of using the HR of 1. In the base case for OS using Coapt we did not assume proportional hazards and modelled each arm independently with exponential models; in sensitivity analysis we assumed proportional hazards holds and applied a HR of 0.62 as reported by Stone et al. [9].

We modelled cumulative hospitalisation by fitting parametric models of hazard to the digitised data: cumulative HFH = {1-H} * max; where H (cumulative hazard) is provided from: (exp(- [h * t]) and hazard (h) is determined by the parametric model expression for hazard, t is month of model, and max represents the plateau value (asymptote) for number of HFHs. This model was then fit to the digitised cumulative HFH using the standard least squares method. Best models were selected on the basis of clinical plausibility and goodness of visual fit. We selected loglogistic models for HFH for both GDMT and Mitraclip arms. This choice is consistent with OS models since extrapolation generates fewer live patients implying a plateau of HFH will be reached at some time point.

### Costs

We hypothesized a full reimbursement of expenses associated with PR (cost index stay + cost of the device). The cost of the Mitraclip system was obtained from the French official journal for the reimbursed indication (as of November 2019) of the medical device in primary severe MR [18]. The cost of the index stay was obtained from the French National Health statistics of hospitalisation (ATIH- *Agence technique de l'information sur l'hospitalisation*). The ATIH was interrogated to identify the most frequently observed Diagnosis-related groups (DRGs) during Year 2019 using the official procedural code DBBF198 associated with PR.

The same methods were used to identify the most frequently observed DRGs following hospitalisation for HF but using International Classification of Diseases (10th revision—ICD-10) codes corresponding to heart failure, namely I500, I501, and I509.

We used monthly costs of medical treatment as reported by Bourguignon et al. [19].

For medical visits, we assumed a frequency of once monthly visit to the general practitioner and twice-yearly visits to the cardiologist. These were valued based on 2019 official fares.

We assumed there were no costs for treating adverse events since in both trials there was no evidence of different safety profile between the two strategies.

Costs included in the economic analysis were considered to be the most up-to-date, so these were not required to be uprated to current prices. Costs (in €) are summarised in Table 1.

**Table 1:** Costs and utilities inputs, and main settings of the model.

Parameters	Base case	Sensitivity analysis	Source for the base case
**Costs**			
Mitraclip device	€21,100.00	+/-20%	French Official Journal 1st March 2018
Mitraclip index stay	€5,398.00		French national DRGs statistics 2019- French Hospital Information System (PMSI/ATIH)
HF Hospitalization	€3,462.80		French national DRGs statistics 2019- PMSI/ATIH using ICD codes I500, I501, and I509
Medical treatment per month	€23.89		Bourguignon et al. [19]
Medical visit at GP practice (once monthly)	€25.00		French official tariff 2019 [20]
Medical visit at cardiologist practice (twice yearly)	€25.00		French official tariff 2019 [20]
**Utilities**			
Stable disease	0.6575	+/-10%	Weighted value based on Griffiths et al. [14]
Disutility for HF hospitalisation	-0.1063		
Disease with hospitalisation	0.5511		
Discount rate	2.50%	0% to 5%	French guidelines [21]
OS model	Exponential	95%CI	Estimation from Iung et al. [10] and Stone et al. [9]
All HF hospitalisations	Loglogistic	+/- 20% on the number of monthly hospitalisations for each arm	
Time Horizon	30 years	5 years	French guidelines [21]
	(lifetime horizon)		

ATIH, Agence technique de l’information sur l’hospitalisation; CI, confidence interval; DRG, diagnosis-related group; GP, general practitioner; HF, heart failure; ICD, international classification of diseases; OS, overall survival.

### Outcomes

We report outcomes as €/QALY gained and €/life year gained.

We obtained utilities for non-hospitalised and hospitalised live states from Griffith et al. [14]. They presented utility values estimated using EuroQol 5D data according to hospitalisation status (no hospitalisation/hospitalisation), then by NHYA class. The utilities for non-hospitalisation and the disutility for HFH for an average patient enrolled in the Mitra-Fr and Coapt trial were obtained by weighting utility values reported in Griffiths et al. [14], so as to reflect the distribution of patients across NYHA classes (Table 1).

### Analysis

We calculated the incremental cost-effectiveness ratio (ICER) as monetary costs (€) per quality-adjusted life-year gained (QALY) and for the Coapt also as € per life-year gained.

A 2.5% annual discount was applied for both costs and benefits [21].

Reimbursement thresholds vary within and between states [22] and Cartier-Bechu et al. 2016 [23] were unable to deduce a reimbursement threshold that operated in France; we therefore arbitrarily adopted €50,000/QALY as the willingness-to-pay (WTP) threshold.

We undertook a one-way deterministic sensitivity analysis for all parameters in order to assess the impact that a fixed change in each parameter has on the ICER (for Tornado diagrams see S3.5 and S4.5 Appendices in S1 File). We applied +/- 20% on costs, 95% upper and lower confidence intervals (UCI and LCI respectively) around parametric models of OS, and +/- 20% on the number of new monthly hospitalisations for HFH.

For multivariate sensitivity analyses, depending on Mitra-Fr or Coapt based economic models, we variously modified influential model inputs identified in univariate sensitivity analysis as follows: a] OS, 95% LCI and UCI for parametric models of OS for each arm; b] +/- 20% on the number of monthly hospitalisations for HFH for each arm; c] +/- 20% on the major costs (consisting of monthly cost of new HFH and the cost of the Mitraclip device); d] +/- 8% or 10% on the utility of the stable disease health state. This procedure generated up to 64 pairs of costs (euros) and benefit (LYG or QALYs) for each arm. These were then used in bootstrapping [24] with 500 iterations to generate the joint distributions of incremental costs and QALYs. The results were graphed using the *Stata ellip* package [25] and used to generate a cost-effectiveness acceptability curves (CEAC).

## Results

### Estimates based on the Mitra-Fr data

Applying an annual 2.5% discount rate in the base case, the deterministic life expectancy for the exponential OS models was 3.96 LYs in both groups consistent with applying a hazard ratio (HR) of 1 for OS (zero incremental LYs).

The undiscounted extrapolated total number of hospitalizations for heart failure over a lifetime horizon was 252 in the intervention group and 328 in the control group, which compares with 159 and 186 respectively within the two-year trial period [10].

Employing the utility values for health states, base case discounted total QALY were almost identical for intervention and the control group resulting in a discounted incremental gain of only 0.00387 QALY.

Total discounted costs were €34,319 and €9,309 for PR+GDMT and GDMT groups respectively, providing an incremental difference between the two strategies of €25,010.

These results provided a base case discounted ICER of €6,467,032 / QALY gained.

One-way deterministic sensitivity analyses (S3.5 Appendix in S1 File) indicate the model is particularly sensitive to changes in OS, to monthly hospitalisation rates and to utility values. Applying an OS HR of 0.76 in favour PR+GDMT over GDMT reduces the ICER to €35,523 / QALY.

The undiscounted life expectancy was 4.38 life years (LYs) for both the intervention and the control groups. Total undiscounted costs were €34,991 and €10,159 for PR+GDMT and GDMT groups respectively, providing an incremental difference between the two strategies of €24,832. The undiscounted ICER was €5,733,897 / QALY.

**Fig 1:**
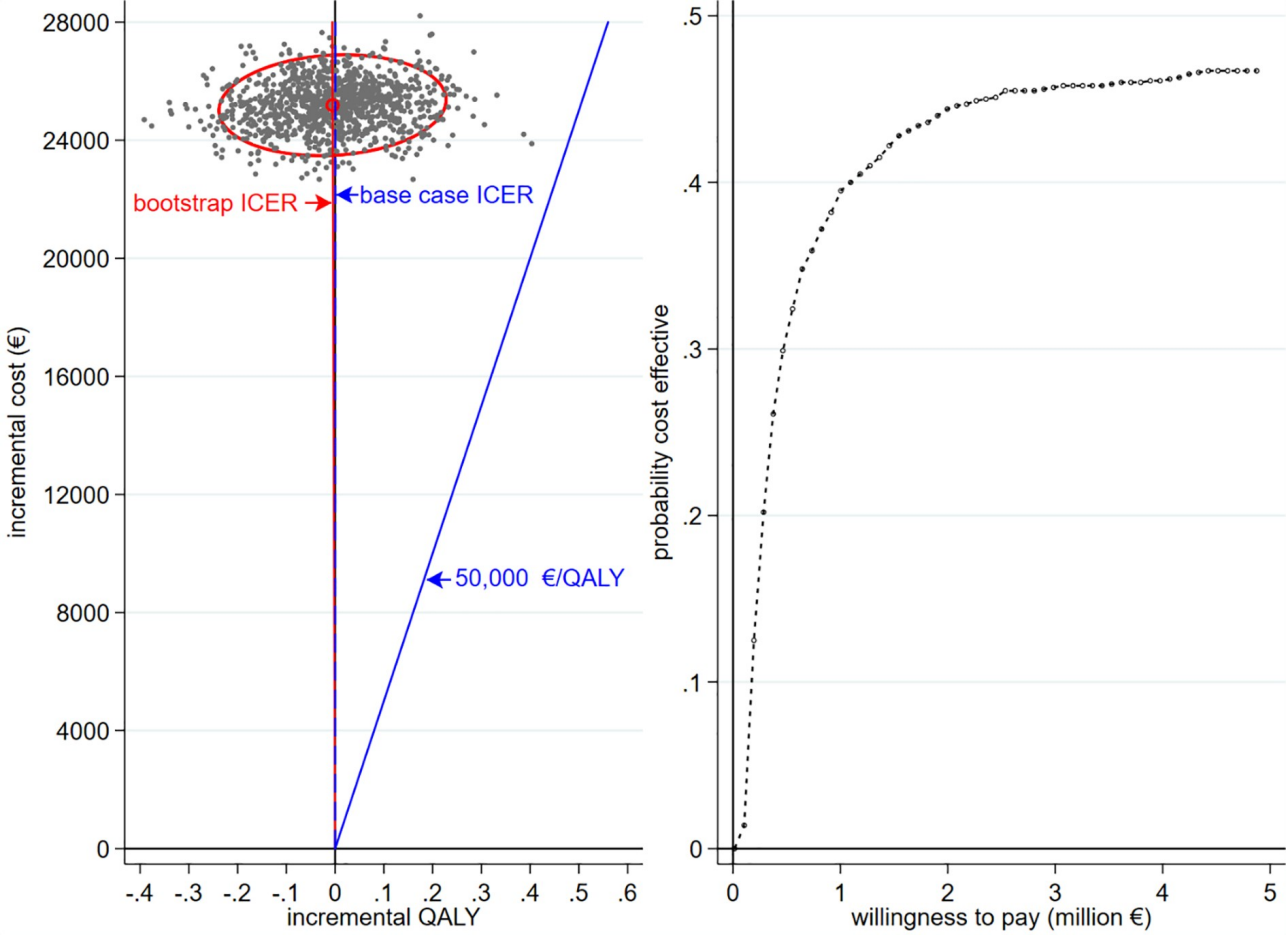
Scatterplot of strategies plotted on an incremental cost-effectiveness plane (a); cost-effectiveness acceptability curve (CEAC) (b), both based on the Mitra-Fr data. In the CE plane the ellipse represents the 95% CI for the 500 means-centred replicates.

In multivariate analysis we varied major costs (+/- 20%), monthly hospitalisations in each arm (+/- 20%) and introduced uncertainty regarding survival gain by using 95% LCI and UCI of the model of GDMT OS as variants for survival in the PR+GDMT arm (equivalent to applying HRs of 0.76 and 1.31). In the resulting scatter plot (Fig 1) just over half (51.8%) of replicates dispersed in the northwest quadrant of the cost-effectiveness plane where PR+GDMT is more costly but yielded fewer QALY than GDMT (i.e. GDMT was dominant). The bootstrap central estimate indicated a loss of 0.0043 QALY (approximately 1.58 quality adjusted days) at an average incremental cost of €5,825,996/QALY. Thus the cost-effectiveness estimate is sensitive to variation in inputs to the model. The CEAC indicates that at a WTP of €50,000 /QALY PR+GDMT has virtually zero probability of being cost effective when compared to GDMT.

### Estimates based on the Coapt data

After discounting, the model generated LYs of 4.93 years and 3.12 years for the PR+GDMT and GDMT groups respectively resulting in an incremental 1.80 LYs.

The extrapolated total number of hospitalizations for HF over a lifetime horizon was 215 in and 345 for PR+GDMT and GDMT respectively, which compares with 160 and 283 respectively within the two year observation period in the RCT [9].

Employing utility values for health states, discounted total QALY in the base case were 3.23 and 2.047 for PR+GDMT and GDMT respectively, resulting in an incremental 1.19 QALY.

Total base case discounted costs were €31,879 and €5,749 for intervention and the control group respectively (incremental of €26,130 between the two strategies), resulting in a base case ICER of €21,918 / QALY gained (discounted value).

**Fig 2:**
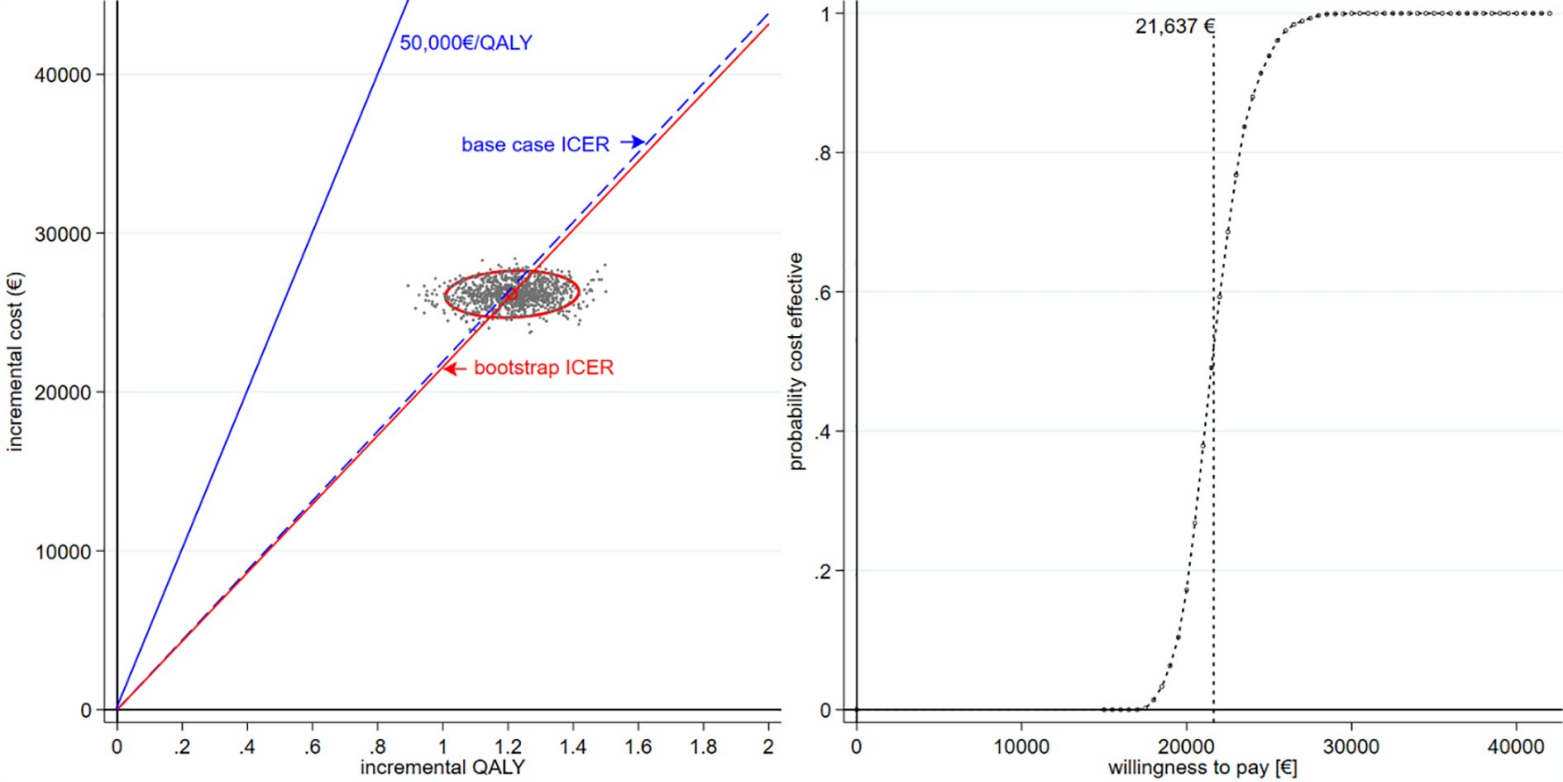
Scatterplot of strategies plotted on an incremental cost-effectiveness plane (a); cost-effectiveness acceptability curve (CEAC) (b), both based on the Coapt data. In the CE plane the ellipse represents the 95% CI for the 500 means-centred replicates.

In multivariate sensitivity analysis, the 500 bootstrap iterations were dispersed in the North West quandrant well below the threshold WTP of €50,000/QALY; the CEAC indicated that at this threshold PR+GDMT had a probability of 1.0 of being cost effective relative to GDMT (Fig 2). The bootstrap ICER of €21,637 / QALY gained differed slightly from the deterministic base-case value of €21,918 / QALY.

One-way sensitivity analyses (S3.5 Appendix in S1 File) indicated the model was reasonably robust but sensitive to changes in time horizon and in OS extrapolations.

The undiscounted life expectancy for the exponential OS models was 5.96 LYs and 3.38 LYs for the intervention and the control group respectively resulting in an incremental 2.21 LYs. Undiscounted total QALY in the base case were 3.67 and 2.21 for PR+GDMT and GDMT respectively, resulting in an incremental 1.46 QALY.

Total undiscounted costs were €32,143 and €6,049 for PR+GDMT and GDMT groups respectively, providing an incremental difference between the two strategies of €26,364.

The undiscounted ICER was €18,064 / QALY.

The Coapt base case analysis assumes that survival benefit seen during 2 years of observation endures for lifetime. We examined the impact on the ICER of curtailing the duration of benefit to between 4 and 18 years, and secondly by allowing the treatment effect to wane linearly from two years to between 6 and 29 years. In these analyses the ICER remains below €50,000 / QALY if the treatment effect endures at least 6 years in the former and at least 10 years in the latter (S3.6 Appendix in S1 File).

Coincident with submission of our analyses 36 month outcome results for Coapt were presented [26]. After 2 years GDMT patients could cross over to PR+GDMT and 38.4% of patients eligible to cross-over did so; the remaining GDMT patients exhibited a notable increase in the rate of hospitalisation for HF infering they were no longer representative of the randomised population. We therefore conducted sensitivity analysis using 3 year data for the PR+GDMT arm and modelled hospitalisation for the GDMT arm by applying the two year HR reported of 0.51 in favour of PR+GDMT (S3.7 Appendix in S1 File). This had little impact on the ICER which was found at €22,119 / QALY.

## Discussion

We have evaluated the cost-effectiveness of PR using two different sources of clinical effectiveness, one from the French Mitra-Fr study, and the other from the US Coapt study.

Analyses based on Mitra-Fr suggest a dramatically high ICER (>€ 6 million/QALY) with sensitivity analyses indicating no potential that PR can be cost-effective in HF patients with severe MR. This result is not unexpected since overall this trial showed no benefit of PR on all hard clinical endpoints at two years [10]. The cost-effectiveness analysis was justified taking into account the observed trend, albeit unconsolidated, towards more favourable results with PR during the second year of follow-up on both the proportion of patients with at least one unplanned HFH and all HFH. Similarly, at two years, the modelled number of HFHs was numerically lower with PR+GDMT than with GDMT alone (159 versus 186 respectively).

In particular, extrapolation suggests benefit in reducing HFHs which translates into lower costs with PR+GDMT; however, this minimally offsets additional costs associated with PR. Indeed, the modelled reduction of HFHs generates a very modest increase QALY which is insufficient to compensate the absence of survival benefit from PR (in Mitra-Fr rates of OS at 2 years were indistinguishable between arms [10]). Moreover, the very small incremental gain estimated with PR (0.00387 QALY, equivalent to 1.41 quality-adjusted days over the lifetime horizon) renders the ICER sensitive to changes applied to some variables and very uncertain.

Employing data from the US Coapt RCT generates strikingly different view since both deterministic and sensitivity analyses suggest PR is good value for money. Interestingly, estimated incremental costs using the US data are very similar compared to those estimated using the French data (€26,130 versus €25,010 respectively). The most striking difference between the two analyses is in the incremental effectiveness expressed as either life-years gained (1.8 with the US data versus 0 with the Mitra-Fr) or QALY gained (1.19 versus 0.00387 respectively).

A few patients in both trials went on to receive further interventions including left ventricular assist device(s) (LVAD, device types unspecified) and or heart transplant. In comparison to medical management LVAD interventions are reported to have relatively high ICERs whether used as destination therapies or as bridge to heart transplant [27–29]. The trajectory of these expensive interventions beyond the randomized phase of the two trials is difficult to model given the lack of data. In common with others [11, 12] we have not included life time costs of these additional interventions.

An economic evaluation from the perspective of the US health care system and conducted alongside the Coapt trial has reported an ICER of US$ 40,361 / life-year gained and US$ 55,600 / QALY gained [11]. The lower ICER obtained in our analysis is partially explained by a lower incremental cost estimated in our work consistent with different costs within health care systems. A higher incremental effectiveness was estimated from our analysis (1.8 LYs versus 1.13 LYs, and 1.19 QALY versus 0.82 QALY). The fact that the US authors have applied a waning of the effect with PR from two years onward likely mostly explains the lower estimated gain in the US analysis.

Our discrepant results from the two cost-effectiveness analyses are mainly due to very different results seen in the incremental effectiveness and are thus consistent with the conflicting effectiveness evidence seen in the Mitra-Fr and the Coapt trials. Although many hypotheses have been suggested to account for trial differences [30–33] it remains unclear which patient categories benefit from PR and which do not, especially as baseline characteristics of enrolled patients are overall similar in the two trials (S2 Appendix in S1 File). Summarizing evidence across studies using meta-analysis of aggregate data appears inappropriate owing to the considerable heterogeneity between studies [34]. Indeed, such heterogeneity cannot be resolved satisfactorily with conventional meta-regression or subgroup analyses techniques.

There may be patient subgroups in the Mitra-Fr trial who derive survival benefit from PR and conversely there may be patient subgroups in the Coapt trial who do not benefit from PR.

In our view, identifying such subgroups represents a major objective. From a clinical viewpoint, this may eventually ensure adequate medical decision making by helping the selection of candidate patients and potentially avoiding futile interventional procedures.

From the economic perspective given the substantial cost of PR, this can result in optimal use of resources. Achieving this objective is also important for regulatory and health technology assessment agencies. Efforts to best reconcile the conflicting findings between the Mitra-Fr trial and the Coapt trials should be strongly encouraged. A promising approach in this direction is possibly the willingness of both steering committees to plan an individual patient data meta-analysis of the two RCTs.

Our work presents several potential limitations. We used reconstructed IPD rather than using actual Mitra-Fr and Coapt trial IPD; however, we used robust methods that generated KM survival plots very closely similar to those published. We used extrapolations beyond observed survival data which must inevitably always be open to question, even though the choice was informed by visual fit, statistical criteria and clinical plausibility. The use of three health states here, as elsewhere, represents an obvious oversimplification of the experience of patients with MR. When compared to two population-based cohort studies on HF patients, our predicted overall survival appears either similar [35] or slightly pessimistic [36]. However, these external comparisons should be viewed cautiously since we were not able to identify published studies specifically reporting long-term survival in HF patients with severe secondary MR. This further contributes to add uncertainty in our extrapolations, which calls for future updates of our cost-effectiveness analyses as longer-term follow-up of both trials may become available.

Last, based on available data, we had to use the same utility inputs throughout the entire model duration. Ideally, utility values would expect to vary with increasing age and increasing number of hospitalisations.

## Conclusion

Cost effectiveness results were conflicting; reconciling differences between trials is a priority and could promote optimal resource allocation.

## Supporting information

S1 File Supplementary appendix. (DOCX)
