# Supplementary material for: Conflicting findings between the Mitra-Fr and the Coapt trials: Implications regarding the cost-effectiveness of percutaneous repair for heart failure patients with severe secondary mitral regurgitation
Source: PLoS One. 2020 Nov 9;15(11):e0241361. doi: 10.1371/journal.pone.0241361 (PMC7652317; doi:10.1371/journal.pone.0241361)
Supplement: S1 File — (DOCX) [file pone.0241361.s001.docx]

S1 FILE. SUPPLEMENTARY APPENDIX

This appendix has been provided by the authors to give readers additional information about their work.

Supplement to: Xavier Armoiry, Jean-François Obadia, Peter Auguste, Martin Connock. Conflicting findings between the Mitra-Fr and the Coapt trials: implications regarding the cost-effectiveness of percutaneous repair for heart failure patients with severe secondary mitral regurgitation

Content

[**S1 Illustrative structure of the model** 3](#_Toc49979144)

[**S2 Comparison of main baseline characteristics and outcomes between the COAPT and MITRA-FR studies** 4](#_Toc49979145)

[**S3 Analyses and results based on data from the Mitra-Fr trial llustrative structure of the model** 6](#_Toc49979146)

[**S3.1 Overall survival Kaplan Meier plots using reconstructed IPD** 6](#_Toc49979147)

[**S3.2 AIC BIC values for parametric models of overall survival** 7](#_Toc49979148)

[**S3.3 Parametric models of overlall survival** 8](#_Toc49979149)

[**S3.4 Parametric models of cumulative hospitalisation** 9](#_Toc49979150)

[**S3.5 Tornado diagram (one way sensitivity analysis) using data from the Mitra-F study** 11](#_Toc49979151)

[**S4 Analysis and results based on data from Coapt trial** 13](#_Toc49979152)

[**S4.1 Overall survival Kaplan Meier plots using reconstructed IPD** 13](#_Toc49979153)

[**S4.2 AIC BIC values for parametric moidels of overall survival** 14](#_Toc49979154)

[**S4.3 Parametric models of overall survival** 15](#_Toc49979155)

[**S4.4 Parametric models of cumulative hospitalisation** 16](#_Toc49979156)

[**S4.5 Tornado diagram (one way sensitivity analysis) using data from the Coapt study** 18](#_Toc49979157)

[**S4.6 Duration of overall survival treatment benefit in Coapt trial** 19](#_Toc49979158)

# **S1 Appendix. Illustrative structure of the model**


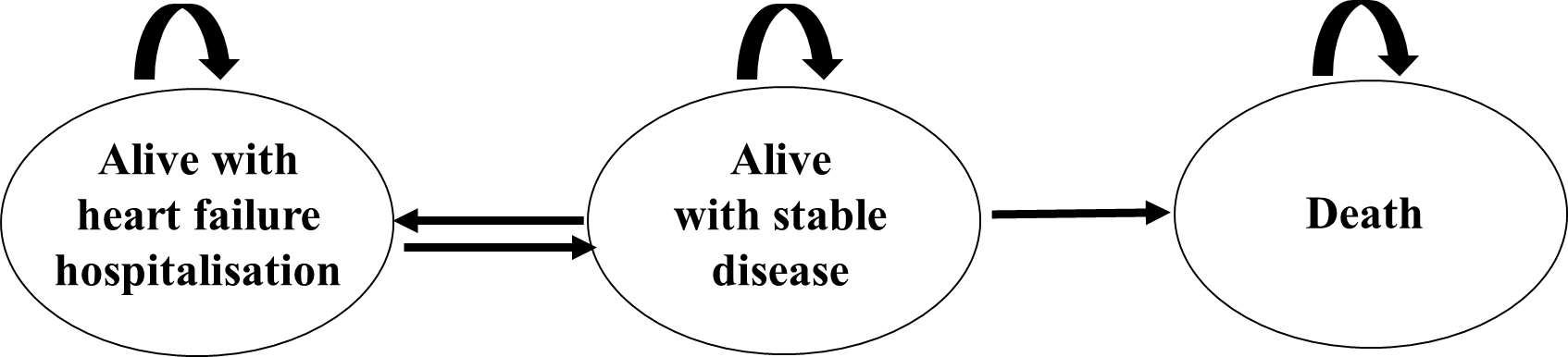


**The Markov type model for PR versus optimal care for severe MR in heart failure.**

The ellipses represent health states, and each arrow represents a possible transition from one state to another. All simulated patients start in the stable disease state before progressing into hospitalization or death, or remaining in the stable state. Patients remain in the hospitalization health state for only 1 month and then return to the stable disease state (i.e. it is assumed they are discharged from hospitalisation in live state).

# **S2 Comparison of main baseline characteristics and outcomes between the COAPT and MITRA-FR studies**

| Study (ref) | COAPT  (Stone et al. NEJM 2018) | |  | Mitra-Fr  (Obadia et al. NEJM 2018, Iung et al. EJHF 2019) | |
| --- | --- | --- | --- | --- | --- |
| Arms | MitraClip + GDMT (n=302) | GDMT alone (n=312) |  | MitraClip + GDMT (n=152) | GDMT alone (n=152) |
| Age (years) | 71.7 ± 11.8 | 72.8 ± 10.5 |  | 70.1±10.1 | 70.6±9.9 |
| Male | 201 (66.6) | 192 (61.5) |  | 120 (78.9) | 107 (70.4) |
| Diabetes | 106 (35.1) | 123 (39.4) |  | 50 (32.9) | 39 (25.7) |
| Previous Myocardial Infarction | 156 (51.7) | 160 (51.3) |  | 75 (49.3) | 52 (34.2) |
| Previous coronary revascularization | 130 (43) | 153 (49) |  | 71 (46.7) | 64 (42.4) |
| Ischemic cardiomyopathy | 184 (60.9) | 189 (60.6) |  | 95 (62.5) | 85 (56.3) |
| Non-ischemic cardiomyopathy | 118 (39.1) | 123 (39.4) |  | 57 (37.5) | 66 (43.7) |
| NYHA class II | 129 (42.7) | 110 (35.4) |  | 56 (36.8) | 44 (28.9) |
| NYHA class III | 154 (51) | 168 (54) |  | 82 (53.9) | 96 (63.2) |
| NYHA class IV | 18 (6) | 33 (10.6) |  | 14 (9.2) | 12 (7.9) |
| HF hosp the year prior inclusion | 176 (58.3) | 175 (56.1) |  | 152 (100) | 152 (100) |
| Atrial Fibrillation | 173 (57.3) | 166 (53.2) |  | 49 (34.5) | 48 (32.7) |
| Left ventricular ejection fraction - % | 31.3 ± 9.1 | 31.3 ± 9.6 |  | 33.3±6.5 | 32.9±6.7 |
| Single implantable cardioverter-defibrillator | 91 (30.1) | 101 (32.4) |  | 48 (31.8) | 57 (37.5) |
| Cardiac resynchronization therapy-defibrillator | 115 (38.1) | 109 (34.9) |  | 46 (30.5) | 35 (23.0) |
| STS replacement sc | 7.8 ± 5.5 | 8.5 ± 6.2 |  | NA | NA |
| Euroscore II, median [IQR] | NA | NA |  | 6.6 [3.5 ; 11.9] | 5.9 [3.4 ; 10.4] |
| MR severity enrollement criteria | Severe secondary mitral regurgitation  (US guidelines): EROA >30 mm2 or RV >45 mL/beat | |  | Severe Secondary Mitral Regurgitation (EU guidelines):  EROA >20 mm2 or RV >30 mL/beat | |
| Effective regurgitant orifice area – cm^2^ | 0.41 ± 0.15 | 0.40 ± 0.15 |  | 0.31±0.10 | 0.31±0.11 |
| Left ventricular end-diastolic volume - ml/m^2^ | 101 ± 34 | |  | 136.2±37.4 | 134.5±33.1 |
|  | | |  |  | |
| CrCl (ml/min) | 50.9 ± 28.5 | 47.8 ± 25.0 |  | 48.8±19.7 | 50.2±20.1 |
| BNP (pg/mL) | 1015 ± 1086 | 1017 ± 1219 |  | 765 [417; 1281] -median IQR | 835 [496; 1258]  -median IQR |
| NT-proBNP (pg/mL) | 5174 ± 6567 | 5944 ± 8438 |  | 3407 [1948; 6790] -median IQR | 3292 [1937; 6343]- median IQR |
|  | | |  |  | |
| **GDMT at baseline:** | | |  |  | |
| Beta-blocker | 275 (91.1) | 280 (89.7) |  | 134 (88.2) | 138 (90.8) |
| ACEi/ARB | 204 (67.6) | 187 (60) |  | 111 (73.0) | 113 (74.3) |
| ARNI | 13 (4.3) | 9 (2.9) |  | 14 (10.0) | 17 (12.1) |
| Mineralocorticoid receptor antagonist | 153 (50.7) | 155 (49.7) |  | 86 (56.6) | 80 (53.0) |
| Diuretic | 270 (89.4) | 277 (88.8) |  | 151 (99.3) | 149 (98.0) |
| Chronic oral anticoagulant | 140 (46.4) | 125 (40.1) |  | 93 (61.2) | 93 (61.2) |
|  | | |  |  | |
| **MitraClip procedure characteristics:** | | |  |  |  |
| MitraClip procedure attempted | 293 (97.0%) | NR |  | 144 (94.7) | NR |
| Clip implanted (MitraClip procedure attempted) | 287 (98.0%) | NR |  | 138 (95.8) | NR |
| - 1 clip implanted | 106 (36.2) | NR |  | 63 (45.7) | NR |
| - 2 clips implanted | 157 (53.6) | NR |  | 62 (44.9) | NR |
| - 3 clips implanted | 23 (7.9) | NR |  | 13 (9.4) | NR |
| - 4 clips implanted | 1 (0.3) | NR |  |  | NR |
|  | | |  |  | |
| **Outcomes at 2 years:** | | |  |  | |
| Death, all-cause | 80 (29.1) | 121 (46.1) |  | 53 (34.9) | 52 (34.2) |
| HR (95%CI) | 0.62 (0.46-0.82) | |  | 1.02 (0.70-1.50) | |
|  | | |  |  | |
| Death or HF hospitalization | 129 (45.7) | 191 (67.9) |  | 97 (63.8) | 102 (67.1) |
| HR (95%CI) | 0.57 (0.45-0.71) | |  | 1.01 (0.77-1.34) | |

**S3 Analyses and results based on data from the Mitra-Fr trial illustrative structure of the model**

## **S3.1 Overall survival Kaplan Meier plots using reconstructed IPD**

*The blue line corresponds to the GDMT arm and the redline to the MitraClip arm.*

## **S3.2 AIC BIC values for parametric models of overall survival**

Control

| Model | Obs | df | AIC | BIC |
| --- | --- | --- | --- | --- |
| Ggamma | 152 | 3 | 327.6212 | 336.6929 |
| exponential | 152 | 1 | 326.3879 | 329.4118 |
| Weibull | 152 | 2 | 326.9772 | 333.025 |
| gompertz | 152 | 2 | 325.6699 | 331.7177 |
| lognormal | 152 | 2 | 325.7199 | 331.7676 |
| loglogistic | 152 | 2 | 326.0957 | 332.1435 |
| AIC, Akaike information criterion; BIC, Bayesian information criterion; df, degrees of freedom; Obs, observation | | | | |

Mitraclip

| Model | Obs | df | AIC | BIC |
| --- | --- | --- | --- | --- |
| Ggamma | 151 | 3 | 340.8404 | 349.8923 |
| exponential | 151 | 1 | 349.2385 | 352.2558 |
| Weibull | 151 | 2 | 345.7147 | 351.7492 |
| gompertz | 151 | 2 | 343.8527 | 349.8873 |
| lognormal | 151 | 2 | 341.3087 | 347.3432 |
| loglogistic | 151 | 2 | 344.4408 | 350.4753 |
| AIC, Akaike information criterion; BIC, Bayesian information criterion; df, degrees of freedom; Obs, observation | | | | |

## **S3.3 Parametric models of overlall survival**

Vertical axis = proportion alive, horizontal axis = months; we =Weibull, ll = loglogistic, go =Gompertz, ex= exponential, ggam = generalised gamma, ln = lognormal; red lines = intervention arm models, blue lines = control arm models.

|  |
| --- |
|  |
|  |

## **S3.4 Parametric models of cumulative hospitalisation**

Loglogistic (LL) and lognormal (LN) models generate occurrence of new hospitalisations up to 3 years and are considered clinically plausible. Exponential models (EX) and Weibull (WE) and other models (not shown) curtail new hospitalisations in the control arm within one year and are judged clinically implausible. Red and black circles represent “observed” cumulative hospitalisations in control and intervention arms respectively. Blue and purple lines represent parametric model fit to “observed” hospitalisation in the GDMT and MitraClip arms respectively.

## **S3.5 Tornado diagram (one way sensitivity analysis) using data from the Mitra-F study**

**
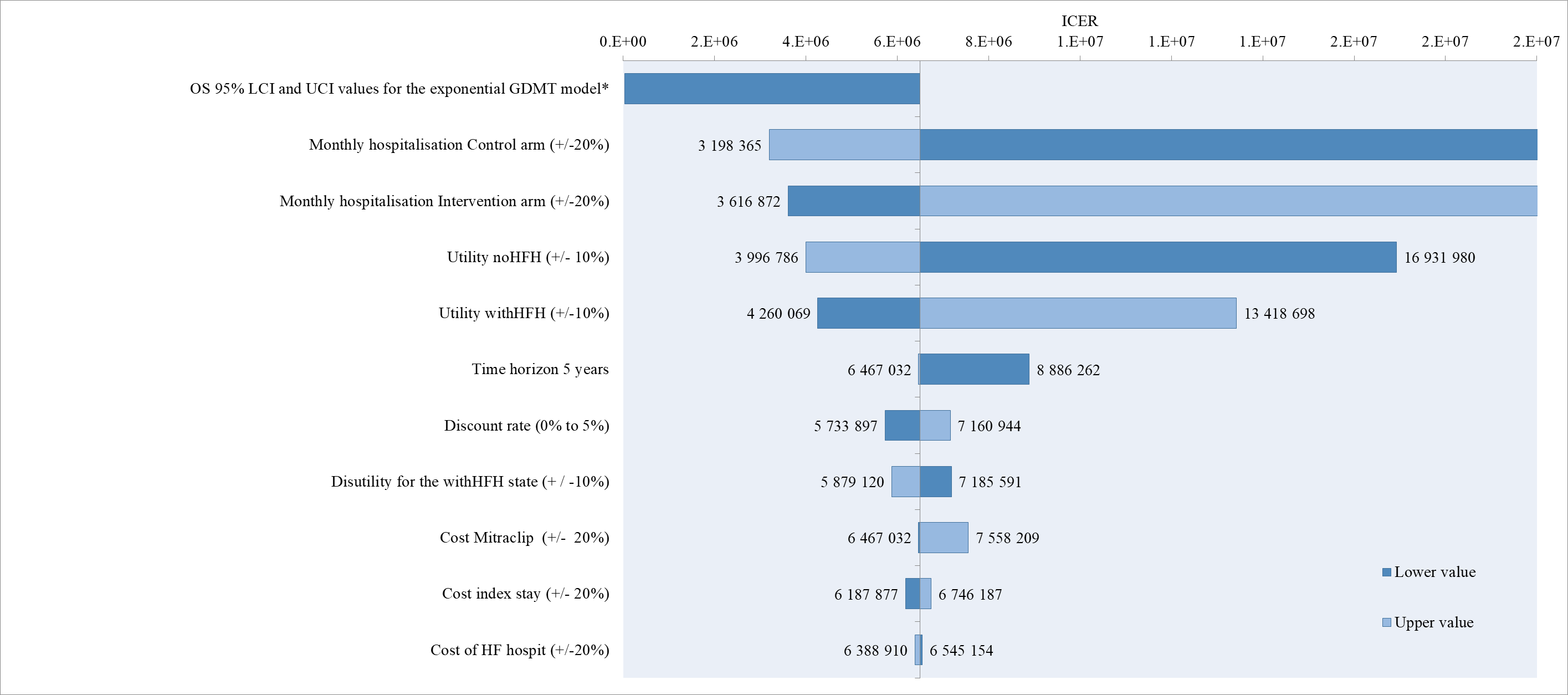
**

* *the corresponding sensitivity analysis is equivalent to applying HRs of 0.76 and 1.31 and shows an ICER reduced to €36,523 / QALY with the lower value. The ICER resulting from applying the upper value has not been presented since the Mitraclip intervention is dominated for any HR above 1.*

**S3.6 Further details on the one way sensitivity analysis to explore the impact of potential improvement in OS with the Mitraclip using data from the Mitra-F study**

| HR applied | ICER |
| --- | --- |
| 1 *(base case)* | 6,468,361 |
| 0.9996 | 5,216,069 |
| 0.999 | 4,041,469 |
| 0.998 | 2,937,550 |
| 0.995 | 1,612,197 |
| 0.990 | 917,682 |
| 0.980 | 490,193 |
| 0.970 | 332,679 |
| 0.960 | 250,770 |
| 0.950 | 200,566 |
| 0.940 | 166,645 |
| 0.930 | 142,191 |
| 0.925 | 132,356 |
| 0.900 | 97,695 |
| 0.850 | 62,659 |
| 0.800 | 45,006 |
| 0.760 *(lower level of the 95%CI for the exponential GDMT model)* | 36,523 |

# **S4 Analysis and results based on data from Coapt trial**

## **S4.1 Overall survival Kaplan Meier plots using reconstructed IPD**

The reported HR was 0.62, the reconstructed HR was 0.60. The black and redlines represent the GDMT and MitraClip arms respectively.

## **S4.2 AIC BIC values for parametric models of overall survival**

Control

| Model | Obs | df | AIC | BIC |
| --- | --- | --- | --- | --- |
| Ggamma | 312 | 3 | 670.3537 | 681.5827 |
| exponential | 312 | 1 | 668.2648 | 672.0078 |
| Weibull | 312 | 2 | 668.6202 | 676.1062 |
| gompertz | 312 | 2 | 669.3828 | 676.8688 |
| lognormal | 312 | 2 | 672.0655 | 679.5515 |
| loglogistic | 312 | 2 | 668.8046 | 676.2906 |
| AIC, Akaike information criterion; BIC, Bayesian information criterion; df, degrees of freedom; Obs, observation | | | | |

Mitraclip

| Model | Obs | df | AIC | BIC |
| --- | --- | --- | --- | --- |
| Ggamma | 302 | 3 | 552.3574 | 563.4887 |
| exponential | 302 | 1 | 551.3696 | 555.0801 |
| Weibull | 302 | 2 | 550.3673 | 557.7881 |
| gompertz | 302 | 2 | 550.5657 | 557.9865 |
| lognormal | 302 | 2 | 554.0978 | 561.5186 |
| loglogistic | 302 | 2 | 550.2766 | 557.6974 |
| AIC, Akaike information criterion; BIC, Bayesian information criterion; df, degrees of freedom; Obs, observation | | | | |

## **S4.3 Parametric models of overall survival**

Vertical axis = proportion alive, horizontal axis = months; we =Weibull, ll = loglogistic, go =Gompertz, ex= exponential, flex2 = flexible parametric, ggam = generalised gamma, ln = lognormal; red lines = intervention arm models, blue lines = control arm models.

|  |
| --- |
|  |
|  |
| **** |

## **S4.4 Parametric models of cumulative hospitalisation**

Loglogistic (LL) and lognormal (LN) models generate occurrence of new hospitalisations up to 3 years and are considered clinically plausible. Exponential models (EX) and Weibull (WE) and other models (not shown) curtail new hospitalisations in the control arm within one year and are judged clinically implausible. Red and black circles reperesent “observed” cumulative hospitalisations in GDMT and MitraClip arms respectively. Blue and purple lines represent parametric model fit to “observed” hospitalisation in the GDMT and MitraClip arms respectively.

## **S4.5 Tornado diagram (one way sensitivity analysis) using data from the Coapt study**


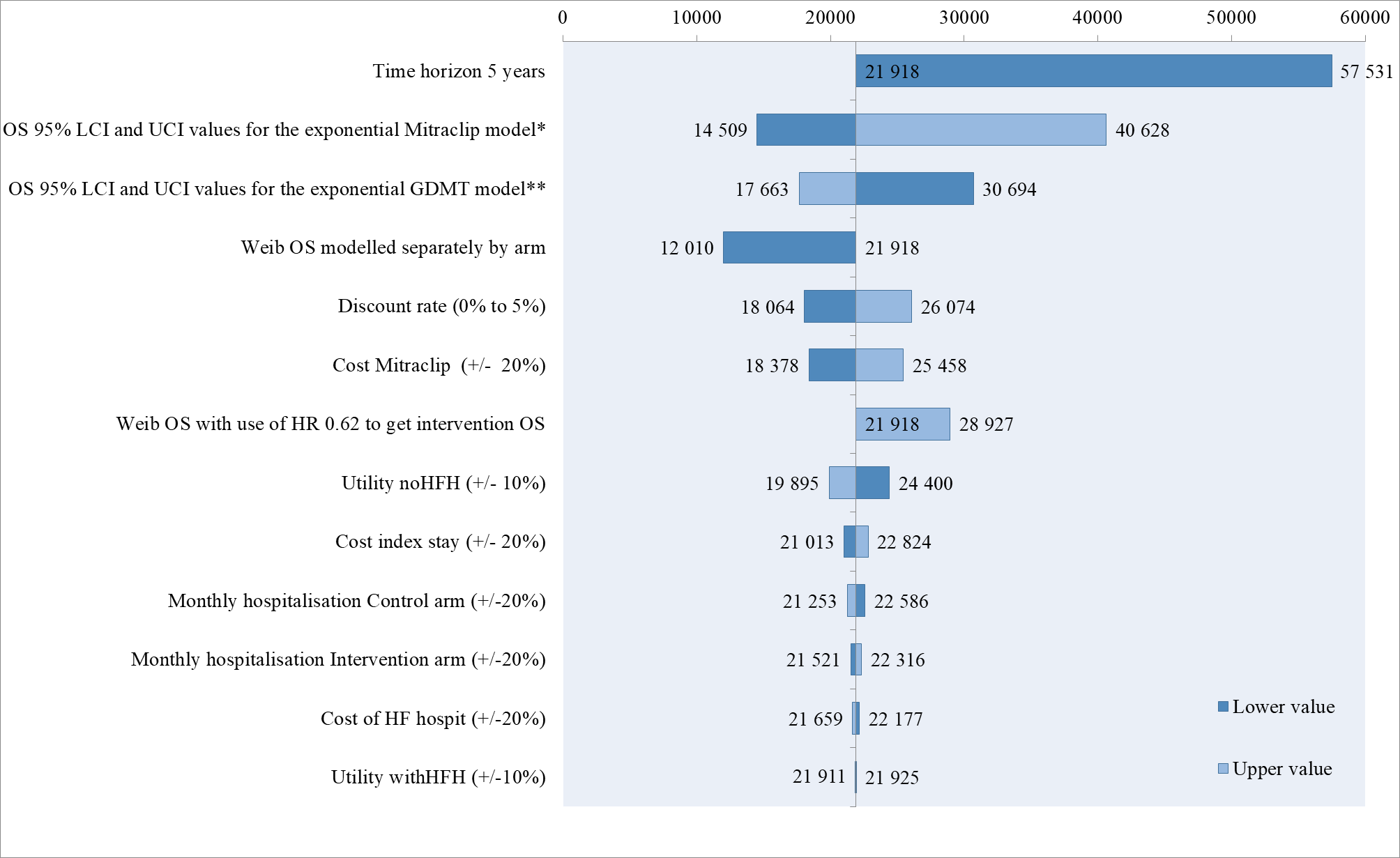


* *the corresponding sensitivity analysis is equivalent to applying HRs of 0.80 and 1.24*

** *the corresponding sensitivity analysis is equivalent to applying HRs of 0.84 and 1.19*

## **S4.6 Duration of overall survival treatment benefit in Coapt trial**

| **A** |  |  |
| --- | --- | --- |
| **B** |  |  |

Considerable survival benefit accrues in the base case economic model under the assumption that the treatment effect is sustained over the life time horizon. To explore the influence of a diminishing treatment effect on the economic model ICER output the duration of the treatment effect was varied in two ways: A] At various times in the model (4 to 18 years) the treatment effect ceases and OS for the Mitraclip arm becomes the same as that for the control arm. B] Starting at two years (end of the observation period) the treatment effect gradually wanes so that the Mitraclip arm OS reaches that of the control arm after various further extensions of effect of 4 years to 27 years.
